# Definition and review of the *lancangjiang* species group of the termitophilous genus *Orthogonius* Macleay, 1825 (Coleoptera, Carabidae, Orthogoniini)

**DOI:** 10.3897/zookeys.349.6164

**Published:** 2013-11-13

**Authors:** Mingyi Tian, Thierry Deuve

**Affiliations:** 1 Department of Entomology, College of Natural Resources and Environment, South China Agricultural University, no. 483, Wushan Road, Guangzhou, 510640, China; 2 Muséum National d’Histoire Naturelle, Département de Systématique & Evolution, UMR 7205 CNRS/Muséum “Organisation, structure et evolution de la biodiversite”, USM 601, Entomologie, Case Postale 50, F–75231 Paris cedex 05, France

**Keywords:** Coleoptera, Carabidae, Orthogoniini, *Orthogonius*, taxonomy, Oriental

## Abstract

The *lancanjiang* species group of the termitophilous ground beetle genus *Orthogonius* Macleay, 1825, is defined and reviewed. This group is characterized by the black and rather elongate body, dense punctation on head and elytra, long and slender appendages, thin fore tibiae, and elytral interval 3 without a subapical setiferous pore. To date, the *lancanjiang* species group is composed of four species and one subspecies, including three new species and one new subspecies which are described in the present paper: *O. macrophthalmus* **sp. n.** (northern Vietnam), *O. euthyphallus* **sp. n.** (southern Vietnam), *O. euthyphallus bolavenensis* **ssp. n.** (southern Laos) and *O. carinatus* **sp. n.** (northern Laos). A distribution map and a key to all species of this group are also provided.

## Introduction

Many species of the termitophilous carabid genus *Orthogonius* Macleay, 1825 have been discovered and recorded since the beginning of this century. This dominant genus of the tribe Orthogoniini is composed of over 200 species and majority of them are distributed in the Oriental Region (Tian and Deuve 2000, 2001, 2003a, b, c, 2004, 2005, 2006a, b, 2007a, b, 2008, 2010; Lorenz 2005; Abhitha et al. 2009; Tian et al. 2012; Tian and Kirschenhofer 2013).

In order to provide information for zoogeographical pattern and phylogeny analysis on the Oriental Orthogoniini, taxonomical treatments on the supra-specific groups of *Orthogonius* are desperately necessary. As a part of the series work, the *lancangjiang* species group is dealt with in the present paper.

*Orthogonius lancangjiang* Tian & Deuve, 2006 was described based on a unique male specimen, from the collection of the Natural History Museum, London. It represents a peculiar lineage in *Orthogonius* by having several character states, *viz*. elongate body and slender appendages, dense punctation on head and elytra, the subapical setiferous pore wanted on interval 3 of elytra, and the male genitalia more or less straight and blunt at apex.

## Materials and methods

All materials used for study were dry and mounted specimens. Dissections, drawings, and observations were made using a binocular Leica MZ75 dissecting microscope. Dissected genital pieces, including the median lobe and parameres of the aedeagus, were glued on small paper cards and then pinned under the specimen from which they were removed. Digital pictures were originally taken with a Canon EOS 40D camera, and then treated by means of CombineZP and Photoshop software. The geographical distribution map was made using Mapinfo Professional 8.5 SCP software.

Length of body is measured from apex of right mandible (in opened position) to apices of elytra. Other abbreviations of measurements in the text are as following:

HL length of head (from apex of right mandible to base of vertex)

HW width of head across eyes

PL length of pronotum along median line

PW greatest width of pronotum

EL length of elytra, from base to apices of elytra along the suture

EW greatest width of combined elytra.

Abbreviations of related museums and collections:

IRSNB Institut Royal des Sciences Naturelle de Belgique, Brussels, Belgium

MNHN Muséum National d’Histoire Naturelle, Paris, France

NHMB Naturalhistorisches Museum, Basel, Switzerland

NHML the Natural History Museum, London, United Kingdom

NHMV Naturalhistorisches Museum, Vienna, Austria

SCAU South China Agricultural University, Guangzhou, China

## Taxonomic treatment

### Diagnostic characters of the *lancangjiang* species group

Members of the *lancangjiang* species group share the following combination of character states: body black and somewhat elongate, appendages slender (Figs 2–9); head and elytra densely punctate; fore tibia thin, outer angle obtuse or disappeared; labrum broadly rounded at front; palps stout, ligula bisetose, each of mentum and submentum bisetose, palpiger asetose; antennae long and slender, extending over basal 2/5 of elytra; pronotum more or less quadrate although both front and hind angles rounded; expanded lateral margins well marked, and slightly reflexed; interval 3 with only fore and middle setiferous pores, the subapical one absent (in the original description of *Orthogonius lancangjiang* Tian & Deuve, 2006, the so-called three pores on interval 3 is actually including a marginal umbilicate pore located near apex of interval 3); elytra well bordered at base; intervals 4–7 normal or partly carinate; elytra broadly truncate, with outer apical angle rounded and inner angle denticulate; middle tibiae not inflate in male, but more slender than in female, and distinctly curved behind middle; prosternal process weakly bordered at apex; middle coxae smooth and glabrous, asetose in the median portion; hind tibiae with apical spurs long, thin and sharp; all tarsal claws distinctly pectinate; hind tarsomere 4 emarginate at apical margin; male genital organ exposed; median lobe of aedeagus quite long, nearly straight on ventral margin, except subapex slightly convex, apex blunt, apical lamella short, tip of apex broadly rounded.

**Sexual dimorphism.** Sexual dimorphism is easily recognized in the *lancangjiang* species group. Although males and females are more or less similar in appearance, they are different on the following aspects: (1) fore tarsomeres 1–3 in male with two rows of spongy setae along median portion (Fig. 10), but lacking in female (Fig. 11); (2) male genitalia (at least apex of the median lobe of aedeagus) more or less exposed; (3) middle tibiae slender and strongly curved inwards in male, while stout and not curved in female; (4) in *Orthogonius carinatus* sp. n. (perhaps also *Orthogonius macrophthalmus* sp. n. in which male is still unknown), elytral intervals 4–6 normal in male, but sub-carinate at bases in female; (5) ventrite VII deeply concave in apical margin in male, while complete in female; (6) antennae slender and a little longer in male than in female; and (7) pronotum finely punctate in male, impunctate in female.

**Figure 1.**
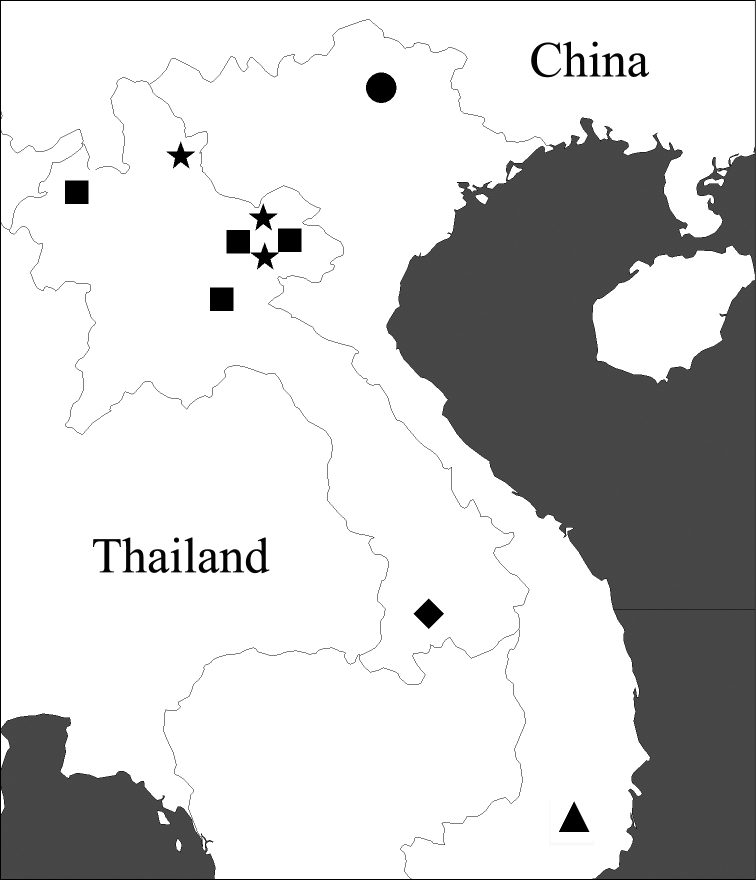
Distribution of the *lancangjiang* species group. ■ *Orthogonius lancangjiang* Tian & Deuve ● *Orthogonius macrophthalmus* sp. n. ★ *Orthogonius carinatus* sp. n. ▲ *Orthogonius euthyphallus* sp. n. ◆ *Orthogonius euthyphallus bolavenensis* ssp. n.

**Geographical distribution of the *lancangjiang* species group.** In total, four species and one subspecies have been recorded in this group. In addition to *Orthogonius lancangjiang*, three species and one subspecies are new to science and described here. The distribution area of the *lancangjiang* species group is limited to Laos and Vietnam according to present data (Fig. 1). It is still uncertain whether or not the Mekong River serves as a distributional boundary in west.

**Figures 2–5.**
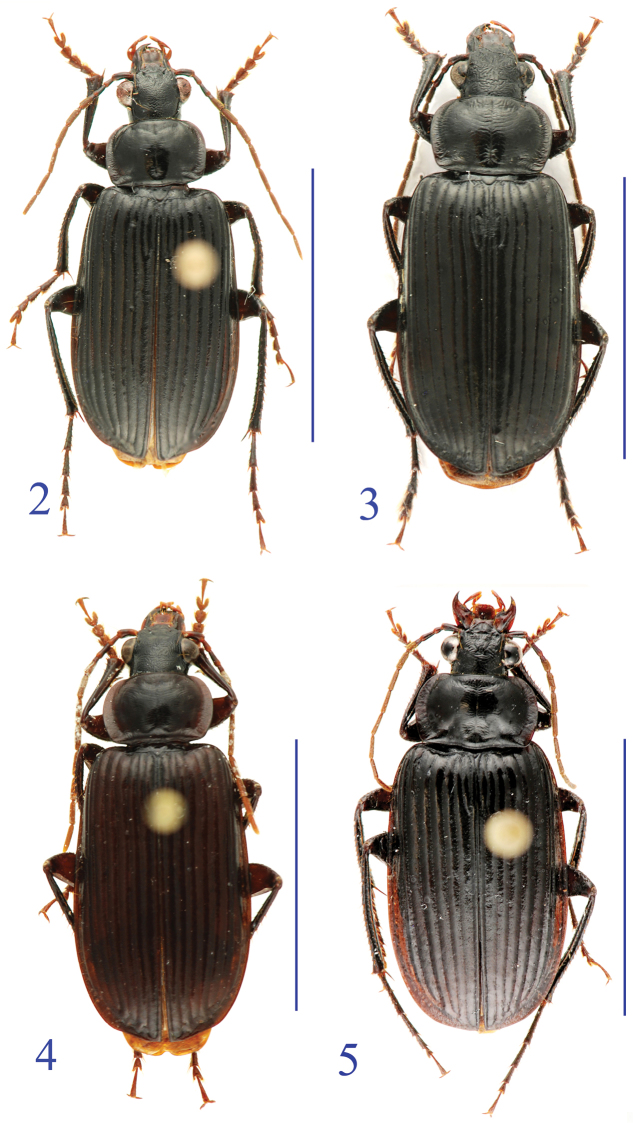
Habitus of the *lancangjiang* species group. **2** *Orthogonius lancangjiang* Tian & Deuve, male **3** idem, female **4** *Orthogonius euthyphallus* sp. n. paratype, male **5** *Orthogonius macrophthalmus* sp. n., holotype, female.

**Figures 6–9.**
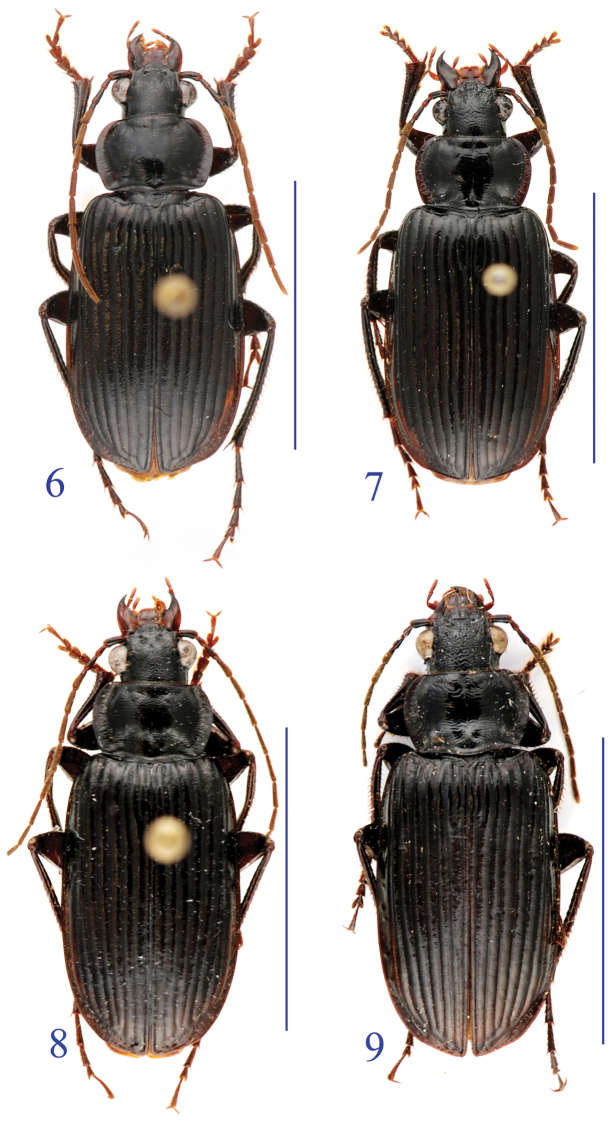
Habitus of the *lancangjiang* species group. **6** *Orthogonius euthyphallus bolavenensis* ssp. n., holotype, male **7** idem, paratype, female **8** *Orthogonius carinatus* sp. n. holoype, male **9** idem, paratype, female.

**Figures 10–11.**
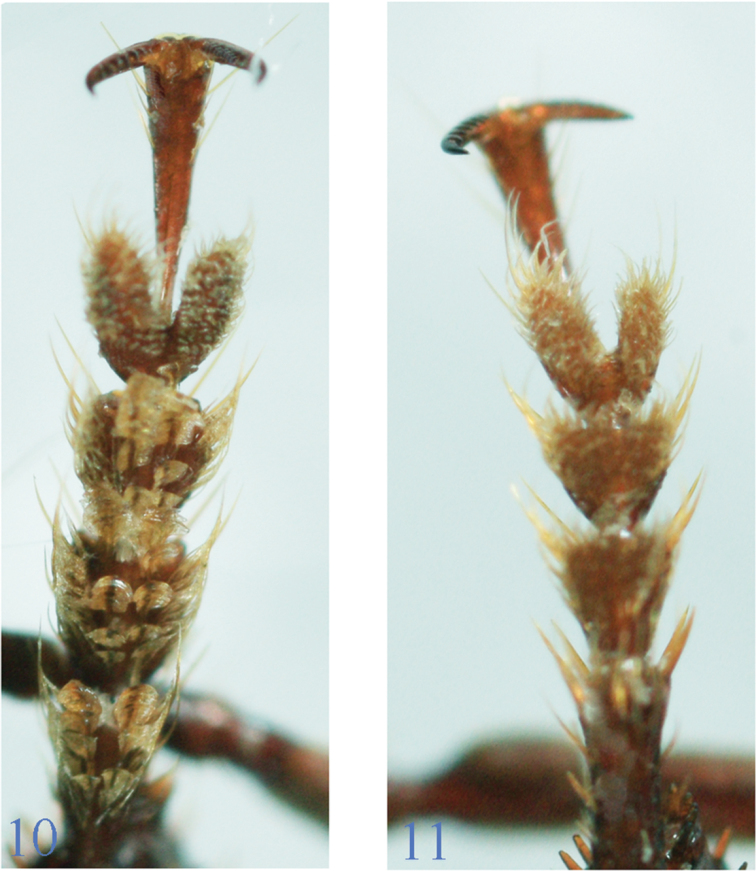
Fore tarsi of *Orthogoniuseuthyphallus* sp. n. **10**, male **11**, Female.

### Key to species of the *lancangjiang* species group

**Table:** 

1	Elytra carinate (at least on interval 7), lateral expanded margin of pronotum distinctly reflexed	2
–	Elytra not carinate, lateral expanded margin of pronotum slightly reflexed	3
2	Eyes very large, strongly prominent, pronotum wider and very transverse (Fig. 5), elytra only carinate on interval 7 in female	*Orthogonius macrophthalmus* sp. n.
–	Eyes moderate in size, less prominent, pronotum narrower and less transverse (Fig. 8), elytra carinate on bases of intervals 4–6 and nearly basal half of interval 7 in female	*Orthogonius carinatus* sp. n.
3	Pronotum covered with denser punctures in male, median lobe of aedeagus (Figs 12–13) nearly straight in profile, the apical lamella stout, nearly obtuse at apex	*Orthogonius lancangjiang* Tian & Deuve
–	Pronotum covered with sparser punctures in male, median lobe of aedeagus (Figs 14–17) more or less widely bisinuate in profile, the apical lamella slender, broad at apex	4
4	Pronotum more transverse, aedeagus indistinctly constricted at subapical portion and the apical lamella narrower in dorsal view (Fig. 15)	*Orthogonius euthyphallus* sp. n.
–	Pronotum broad and less transverse, aedeagus distinctly constricted at subapical portion and the apical lamella broader in dorsal view (Fig. 17)	*Orthogonius euthyphallus bolavenensis* ssp. n.

### Orthogonius lancangjiang

Tian & Deuve, 2006

http://species-id.net/wiki/Orthogonius_lancangjiang

Figs 1 –3 , 12–13

Orthogonius lancangjiang Tian & Deuve, 2006: 134

#### Diagnosis.

Medium to large sized, elytra slender or elongate ovate; head longer than wide; upper surface covered with dense punctures; antennae long; eyes moderate for orthogoniines; labrum round at front; pronotum quite narrow, but transverse in form; whole lateral expanded margins slightly reflexed; elytra not carinate on interval 7 in both sexes.

Length: 12.5–16.0 mm; width (=EW): 5.2–6.5 mm.

#### Description.

Body elongate, strongly shiny. Habitus as in Figs 2–3.

Head and disc of pronotum black; elytra black to dark brown; lateral expanded margins of pronotum, antennomere 1 and 3, mandibles, and tibiae dark brown; other parts brown.

Macrosculpture: Surface of head, pronotum and elytra (including all intervals) with dense punctures, head obscurely striate, pronotum impunctate in most specimens, or with a few faint punctures in one or two individuals, moderately striate or not; underside surface smooth and glabrous, impunctate.

Microsculpture: Engraved meshes densely isodiametric or slightly transverse on head, pronotum and elytra.

Head longer than wide, HL/HW=1.15–1.20, eyes small but prominent, frons and vertex convex, frontal impressions large and deep; clypeus bisetose, basal portion unevenly convex; labrum broad at apical margin, sexsetose; palps rather stout, subcylindrical, maxillary palpomere 3 as long as 4, palpomere 4 glabrous, palpomere 3 with two short setae at apex; labial palpomere 2 slightly longer than 3, bisetose on inner margin, with several additional setae at subapex and apex, palpomere 3 with a few tiny setae; ligula small, bisetose at apex; mentum without tooth, each of mentum and submentum bisetose, palpiger asetose. Antennae very long, extending to middle of elytra; pubescent and slightly expanded from basal 1/3 of antennomere 4; antennomere 3 almost as long as 4, and 1.40 times longer than 2; antennomere 1 unisetose on subapex.

Pronotum strongly transverse, PW/PL=1.56–1.60, disc moderately convex, fore and basal margins well beaded, sides evenly expanded, widest at about middle; lateral expanded margin well defined, flat, very smooth and hardly reflexed; both transversal impressions distinct, basal foveae small and deep.

Elytra elongate ovate, EL/EW=1.45–1.77, widest at about middle, sides slightly expanded at middle, base completely bordered, apex broadly sinuate, inner angle nearly rectangular, obtuse; striae very deep, intervals strongly convex, subequal in width in middle, interval 3 with basal and middle setiferous pores, the subapical one absent.

Legs slender, fore tibia distinctly expanded at apex, apex obliquely truncate, outer margin slightly subserrate; middle and hind coxae smooth and glabrous; middle tibia slender and slightly expanded at apex, strongly curved in male, normal in female; hind femora with two setae posteriorly; hind tibia slightly expanded at apex, apical spurs long and sharp; fore tarsi slightly wider than the middle, and distinctly wider than the hind ones; hind tarsomeres 1 and 3 much longer than 2 and 4 respectively, length ratio of tarsomeres1 to 4 as 3.2: 1.9: 1.5: 1.0; tarsomere 4 asymmetrically emarginated at apex, longer lobe as long as 1/3 of the whole joint; all tarsal claws strongly pectinate.

Prosternal process well bordered at apex, abdominal ventrite VII of male distinctly and deeply emarginate at apical margin.

**Figures 12–15.**
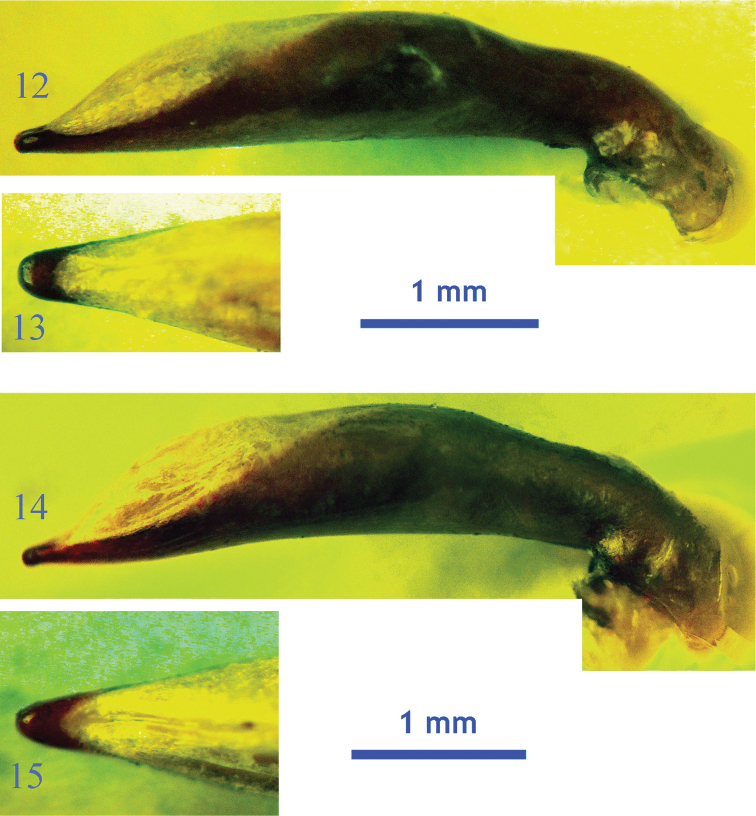
Male genitalia of the *lancangjiang* species group **12** *Orthogonius lancangjiang*, lateral view **13** idem, dorsal view of apical portion **14** *Orthogonius euthyphallus* sp. n., lateral view **15** idem, dorsal view of apical portion.

Male genitalia (Figs 12–13): Slender and quite straight, slightly sinuate on ventral surface, apex blunt, the apical lamella as long as wide, broad at tip.

**Female.** Similar to male, except the abdominal ventrite VII complete at apical margin, fore tarsomeres 1–3 without spongy setae ventrally, middle tibiae not curved in middle portion, pronotum impunctate, and antennae slightly shorter.

#### Variability.

Body slender or a little stouter, in general pronotum impunctate in female, but one or two individuals with faint and sparse punctures.

#### Materials examined.

1 male, the holotype, “Haut Mekong, Vien Poukha, 3. V. 1918, R. V. de Salvaza”, “*Orthogonius* sp.” (by Andrewes) and “Brit. Mus. 1921–89”, from Laos, deposited in NHML; 8 males and 7 females, “Laos-NE: Xieng Khouang Province, Phonsaven (30 km NE), Phou Sane Mt., 19.37–38N/ 103.20E, 1400–1500 m, 10.–30.V. 2009, Z. Kraus Leg.”, and “NHMB Basel, NMPC Prague Laos 2009 Expedition: M. Brancucci, M. Geiser, Z. Kraus, D. Hauck, V. Kuban”; 6 males and 4 females, “Laos-NE: Xieng Khouang Province, Phonsaven (30 km NE), Phou Sane Mt., 19.37–38N/ 103.20–21E, 1400–1700 m, 10.–30.V. 2009, D. Hauck Leg.”, and “NHMB Basel, NMPC Prague Laos 2009 Expedition: M. Brancucci, M. Geiser, Z. Kraus, D. Hauck, V. Kuban”; 7 males and 8 females, “Laos–NE: Xieng Khouang Province, 30 km NE Phonsaven, Phou Sane Mt., 19.37–8N/ 103.20–21E, 1420 m, 30 km NE Phonsaven, Ban Na Lam to Phou Sane Mt., 1300–1700 m, 10.–30.V. 2009, M. Geiser Leg.”, and “NHMB Basel, NMPC Prague Laos 2009 Expedition: M. Brancucci, M. Geiser, Z. Kraus, D. Hauck, V. Kuban”; 1 male, “Laos–NE: Xieng Khouang Province, 30 km NE Phonsaven, Phou Sane Mt., 19.3820N/ 103.2020E, 1420 m, 10.–30.V. 2009, D. Hauck Leg.”, and “NHMB Basel, NMPC Prague Laos 2009 Expedition: M. Brancucci, M. Geiser, Z. Kraus, D. Hauck, V. Kuban”; 7 male 4 females, “Laos–NE: Xieng Khouang Province, 30 km NE Phonsaven: Ban Na Lam to Phou Sane Mt., 19.37–38N/ 103.20E, 1300–1500 m, 10.–30.V. 2009, M. Brancucci Leg.”, and “NHMB Basel, NMPC Prague Laos 2009 Expedition: M. Brancucci, M. Geiser, Z. Kraus, D. Hauck, V. Kuban”; 1 male, “Laos–NE: Houa Phan Province, Ban Saluei to Phou Pane Mt., 20.11–13N/ 103.59–104.01E, 1300–1900 m, 9–17.VI. 2009, Michael Geiser Leg.”, and “NHMB Basel, NMPC Prague Laos 2009 Expedition: M. Brancucci, M. Geiser, Z. Kraus, D. Hauck, V. Kuban”; 2 males and 1 female, “Laos–NE: Houa Phan Province, Ban Saluei to Phou Pane Mt., 20.12–13N/ 103.595–104.01E, 1340–1870 m, 10. V.–16. VI. 2009, M. Brancucci and local coll. Leg.”, and “NHMB Basel, NMPC Prague Laos 2009 Expedition: M. Brancucci, M. Geiser, Z. Kraus, D. Hauck, V. Kuban”; 2 males, “Laos-NE: Houa Phan Province, Ban Saluei to Phou Pane Mt., 20.13N/ 104.00E, 1350–1500 m, 1–16. VI. 2009, M. Brancucci Leg.”, and “NHMB Basel, NMPC Prague Laos 2009 Expedition: M. Brancucci, M. Geiser, Z. Kraus, D. Hauck, V. Kuban”; 8 females, “Laos–NE: Houa Phan Province, Ban Saluei to Phou Pane Mt., 20.12N/ 104.01E, 1500–1900 m, 17.V.–3.VI. 2007, M. Brancucci Leg.”, and “NHMB Basel Expeditionto Laos, 2007”. All in NHMB, except for eight males and eight females in MNHN and SCAU, respectively.

#### Distribution.

Laos (Fig. 1).

### Orthogonius euthyphallus

Tian & Deuve sp. n.

http://zoobank.org/596B1C35-4776-4C08-8D97-CD945B431249

http://species-id.net/wiki/Orthogonius_euthyphallus

Figs 1 , 4 , 10–11 , 14–15

#### Diagnosis.

Medium to large sized, slender and elongate; head longer than wide; upper surface covered with dense punctures; antennae long; eyes moderate in size; labrum broad at front margin; pronotum quite narrow, but transverse in form; whole lateral expanded margins slightly reflexed; elytra not carinate on interval 7 in both sexes.

Length: 14.0–16.0 mm; width: 5.5–6.5 mm. Habitus as in Fig. 4.

#### Description.

Body elongate, strongly shiny.

Head and disc of pronotum black; elytra dark brown or brown; lateral expanded margins of pronotum, antennomere 1 and 3, mandibles, and tibiae dark brown; palps, legs excluding tibiae and antennae brown.

Macrosculpture: Surface of head, pronotum and elytra with dense punctures, head obscurely striate, pronotum and elytra without wrinkles.

Microsculpture: Engraved meshes isodiametric on head and elytra, but moderate transverse on pronotum.

Head longer than wide, HL/HW=1.15–1.18, eyes rather small but prominent, frons and vertex convex, frontal impressions large and deep; clypeus bisetose, basal portion unevenly convex; labrum broad at front margin, sexsetose; palps rather stout, subcylindrical, maxillary palpomere 3 as long as 4, palpomere 4 glabrous, palpomere 3 with two short setae at apex; labial palpomere 2 slightly longer than 3, bisetose on inner margin, with several additional setae at subapex and apex, palpomere 3 bearing a few tiny setae; ligula small, bisetose at apex; mentum without tooth, each of mentum and submentum bisetose, palpiger asetose. Antennae very long, extending to the middle of elytra; pubescent and slightly expanded from basal 1/3 of antennomere 4; antennomrere 3 almost as long as 4, and 1.32 times longer than 2; antennomere 1 unisetoae on subapex.

Pronotum strongly transverse, PW/PL=1.55–1.63, disc moderately convex, fore and basal margins well beaded, sides evenly expanded, widest at about middle; lateral expanded margin well defined, flat and very smooth, slightly reflexed; both transversal impressions distinct, basal foveae small and deep; median line clear.

Elytra elongate ovate, EL/EW=1.71–1.82; widest at a little behind middle, sides slightly expanded at middle, basal border complete, apex broadly sinuate, inner angle nearly rectangular and obtuse; striae very deep, intervals strongly convex, subequal in width in middle, interval 3 with basal and middle setiferous pores, the subapical one absent.

Legs slender, fore tibia distinctly expanded at apex, apex obliquely truncate, outer margin slightly subserrate; middle and hind coxae smooth and glabrous; middle tibia strongly curve, slightly expanded at apex; hind tibia hardly expanded at apex, apical spurs long and sharp, hind tarsomere 3 much longer than 4, length ratio of tarsomeres 1–4 as 2.55, 1.79, 1.32 and 1.0; tarsomere 4 asymmetrically emarginated at apex, longer lobe as long as 1/3 of the whole joint; hind femur thin, slightly expanded medially, with two long setae posteriorly; tarsal claws strongly pectinate.

Prosternal process well bordered at apex, abdominal ventrite VII of male distinctly and deeply emarginate at apical margin.

Male genitalia (Figs 14–15): Slender and quite straight, slightly sinuate on ventral surface, apex blunt; the apical lamella as long as wide, broad at tip.

#### Remarks.

This new species differs from *Orthogonius lancangjiang* by the wider emargination on ventrite VII in males, which is gradually narrowed towards base (suddenly but somewhat obliquely narrowed towards base in *Orthogonius lancangjiang*), the median lobe of aedeagus is less straight, more sinuate ventrally, and apex thin and broad (thick and obtuse at apex in *Orthogonius lancangjiang*); and elytra with sparser punctures (densely punctate in *Orthogonius lancangjiang*).

#### Material examined.

Holotype: male, “S. Vietnam, 28–30.4.1994, 12 km N. Dalat, Lang Bian, Pacholatko & Dembicky”, “Mus. Wien”, in NHMV.

Paratypes. 1 male, idem; 9 males and 3 females, “S. Vietnam, 17–21.4.1995, 12 km N. Dalat, Lang Bian”, “12.03N108.27E, 1580–1750 m, Pacholatko & Dembicky”, and “Mus. Wien”, all in NHMV except 1 male and 1 female in SCAU.

#### Etymology.

The name of the new species is combined by the Greek prefix “*euthy*-”, meaning straight, and word “*phallus*”, meaning penis, to refer to the straight median lobe of aedeagus.

#### Distribution.

Southern Vietnam (Fig. 1).

### Orthogonius euthyphallus bolavenensis

Tian & Deuve ssp. n.

http://zoobank.org/10A4E8EF-DC6B-48A9-AB91-0C1D3D75FE41

http://species-id.net/wiki/Orthogonius_euthyphallus_bolavenensis

**Figures 16–19.**
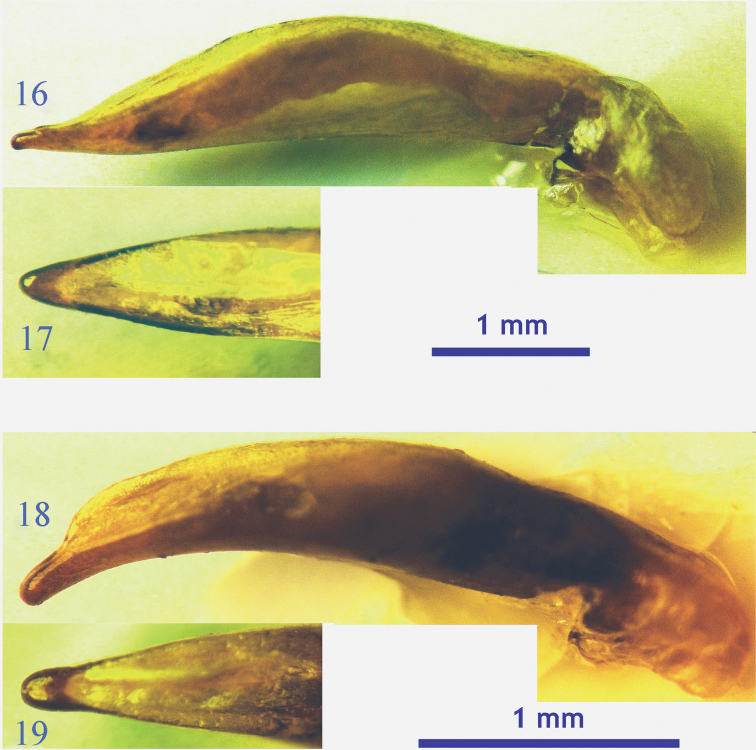
Male genitalia of the *lancangjiang* species group. **16** *Orthogonius euthyphallus bolavenensis* ssp. n., lateral view **17** idem, dorsal view of apical portion **18** *Orthogonius carinatus* sp. n., lateral view **19** idem, dorsal view of apical portion.

Figs 1 , 6–7 , 16–17

#### Description.

Body stout, strongly shiny. Habitus as in Figs 6–7.

Length: 16.5 mm; width: 6.5 mm.

Black, antennomeres 4–11, mandibles, palps and tarsomeres dark brown to brown.

Macrosculpture: Head densely punctured in both sexes, elytra densely punctured in male, (rather sparser in female); pronotum much less punctured in male, without puncture in female; head obscure striate on frons, smooth on vertex; pronotum faintly striate or not; underside surface smooth and glabrous, impunctate.

Microsculpture: Engraved meshes densely isodiametric on elytra, faint on head and pronotum.

Head longer than wide, HL/HW=1.17–1.18, eyes large and prominent, frons and vertex convex, frontal impressions large and deep, extending on clypeus; clypeus bisetose, basal portion deeply furrowed; labrum slightly broad at apical margin, sexsetose; palps rather stout, subcylindrical, maxillary palpomere 3 as long as 4 which is glabrous, palpomere 3 with two short setae at apex; labial palpomere 2 longer than 3, bisetose on inner margin, with several additional short setae at subapex and apex, palpomere 3 with a few tiny setae; ligula small, bisetose at apex; mentum without tooth, each of mentum and submentum bisetose, palpiger asetose. Antennae rather stout, not extending over the middle of elytra; pubescent from basal 1/4 of antennomere 4; antennomere 3 almost as long as 4, and 1.55 times longer than 2; antennomere 1 unisetose on subapex.

Pronotum rather broad though transverse, PW/PL=1.49–1.52, disc moderately convex, fore and basal margins well beaded, sides evenly expanded, widest at about middle; lateral expanded margin wide and hardly reflexed, better defined in female than in male; both transversal impressions indistinct, basal foveae shallow, median line clear.

Elytra elongate ovate, EL/EW=1.57–1.60, widest at about middle, where sides slightly expanded, base completely bordered, apex broadly sinuate, inner angle nearly rectangular, faintly denticulate; striae deep, intervals convex, subequal in width in middle portion, interval 3 with basal and middle setiferous pores, the subapical one absent.

Legs slender, fore tibia distinctly expanded at apex, apex obliquely truncate, outer margin smooth, not subserrate; middle and hind coxae smooth and glabrous in median portion; middle tibia slender and slightly expanded at apex, strongly curved in male, while normal in female; hind femur moderately expanded, with two setae posteriorly; hind tibia slightly expanded at apex, apical spurs long and sharp; fore tarsi distinctly wider than middle and hind ones, middle wider than the hind; hind tarsomeres 1 and 3 much longer than 2 and 4 respectively, length ratio of tarsomeres 1–4 as 2.57, 1.86, 1.28 and 1.0; tarsomere 4 asymmetrically emarginat, with longer lobe as deep as 1/3 of the joint; all tarsal claws strongly pectinate.

Prosternal process well bordered at apex, abdominal ventrite VII of male narrowly and deeply emarginate at apical margin.

Male genitalia (Figs 16–17): Slender and quite straight, expanded medially, gently sinuate on ventral margin, apex stout and blunt in profile; in dorsal view, broad at subapical portion, the apical lamella slightly wider than long, broad at tip.

#### Remarks.

Similar to the nominate subspecies, but darker and broader (in particular pronotum and elytra), and the apical lamella is more slender in dorsal view.

#### Material examined.

Holotype: male, “Coll. I. R. Sc.N.B/ purchased from Mr. B. Makovsky, I.G. 31.969” and “Laos South, Bolaven Plateau, Ban Itou, 23.–27. V. 2007, B. Makovsky Lat.”, in IRSNB.

Paratype. 1 female, idem, in IRSNB.

#### Etymology.

Refers to its type locality.

#### Distribution.

Southern Laos. Known only from the type locality (Fig. 1).

### Orthogonius carinatus

Tian & Deuve sp. n.

http://zoobank.org/55C5C2C1-598B-4F4C-98D9-50B26CA66935

http://species-id.net/wiki/Orthogonius_carinatus

Figs 1 , 8–9 , 18–19

#### Diagnosis.

Medium to large sized, body elongate, whole dorsal surface extraordinarily and densely punctate; head as long as wide, eyes moderate, labrum roundly protruding at front; pronotum narrow, lateral expanded margins distinctly reflexed throughout; elytra carinate on basal 1/3 of interval 7 in both sexes, and at base of intervals 4–6 in female, but normal in male.

Length: 14.0–16 mm; width: 5.5–6.0 mm. Body slender and elongate. Habitus as in Figs 8–9.

#### Description.

Black, antennomeres 1, 3–4, legs (except tarsomeres) and ventral surface dark brown, mandibles, labrum, palps, other antennomeres and tarsomeres reddish brown.

Macrosculpture: Head extraordinarily and densely punctate, pronotum distinctly punctate in male, but faintly punctate or impunctate in female; elytra with tiny, sparse and faint punctures in both sexes; underside smooth and glabrous.

Microsculpture: Engraved meshes densely isodiametric or slightly transverse on elytra, faint or not clear on head and pronotum.

Head stout, as long as wide,eyes moderate, prominent, frons and vertex moderately convex, frontal impressions not well defined, shallow; clypeus bisetose, base area rugose; labrum broadly protruding at front, sexsetose; mandibles rather short; ligula broad, bisetose at apex; maxillary palps moderate, subcylindrical, maxillary palpomere 3 slightly longer than 4, both glabrous except for palpomere 3 with two short setae at apex; labial palpomeres stout, palpomere 3 somewhat expanded, palpomere 2 cylindrical, as long as palpomere 3, bisetose on inner margin, and with two or three additional setae at subapex and apex; palpiger asetose, mentum and submentum each with one pair of setae; mentum without median tooth. Antennae long and slender, extending beyond basal 1/3 of elytra; pubescent from basal 1/3 of antennomere 4, antennomere 3 slightly shorter than 4.

Pronotum transverse, PW/PL=1.55–1.60, disc moderately convex; sides evenly rounded, widest at middle, both basal and fore margins beaded, lateral expanded margins well defined, wide and obviously reflexed throughout; fore and hind angles rounded; both fore and hind transversal impressions faint, basal foveae large and wide, connected to expanded margins; median line clear.

Elytra elongate, EL/EW=1.72–1.78, moderately convex, base completely bordered; sides nearly parallel at middle, striae deep, intervals convex, subequal in width in middle; apex broadly truncate; carinate on bases of intervals 6–8, basal 1/3 of interval 7 in both sexes, and at base of intervals 4–6 in female (carination shortest on interval 4, and longest on 6); interval 3 with only the basal and middle setiferous pores, the subapical one absent.

Legs slender; middle and hind coxae glabrous in median portion; fore tibia slightly dilated at apex, outer angle obtuse, apex slightly and obliquely truncate, outer margin slightly subserrate; middle and hind tibiae hardly dilated at apex, slender and strongly curved in middle portion in male, normal in female; hind tarsomeres 1 and 3 much longer than 2 and 4 respectively, length ratio of tarsomeres 1–4 as 3.9: 2.6: 1.8: 1.0; tarsomere 4 deeply emarginate at apex, lobes asymmetric, longer lobe as long as 1/3 of the joint; all tarsal claws strongly pectinate.

Prosternal process faintly bordered at apex. Abdominal ventrite VII distinctly emarginate at apical margin in male.

Male genitalia (Figs 18–19). Rather slender, strongly sinuate ventrally, distinctly arcuate at apical 1/3, slightly dilated in middle portion, dorsal opening large, subapex nearly paralleled towards apex, broadly obtused at apex; in dorsal view, apical lamella almost as long as wide, not parallel–sided, apex broadly rounded.

#### Remarks.

Similar to *Orthogonius lancangjiang*, but intervals of elytra carinate; the median lobe of aedeagus distinctly arcuate and the apical lamella somewhat stouter. It is easily distinguished from the next species, *Orthogonius macrophthalmus* sp. n., by its small and less prominent eyes, narrower pronotum, and elytra carinate on base of intervals 4–6 in female.

#### Material examined.

Holotype: male, labeled: “Laos–NE: Houa Phan Province, Ban Saluei to Phou Pane Mt., 20.12 N/ 104.01 E, 1500–1900 m, 17.V.–3.VI. 2007, M. Brancucci Leg.”, and “NHMB Basel Expeditionto Laos, 2007”, in NHMB.

Paratypes. 1 male and 3 female, idem; 1 male 3 females, “Laos–NE: Houa Phan Province, Ban Saluei to Phou Pane Mt., 20.1309N/ 103.5954–104.0003E, 1480–1550 m, 9–16. VI. 2009, David Hauck Leg.”, and “NHMB Basel, NMPC Prague Laos 2009 Expedition: M. Brancucci, M. Geiser, Z. Kraus, D. Hauck, V. Kuban”; 1 male and 5 females, “Laos–NE: Xieng Khouang Province, 30 km NE Phonsaven, Phou Sane Mt., 19.37–8N/ 103.20–21E, 1420 m, 30 km NE Phonsaven, Ban Na Lam to Phou Sane Mt., 1300–1700 m, 10.–30.V. 2009, M. Geiser Leg.”, and “NHMB Basel, NMPC Prague Laos 2009 Expedition: M. Brancucci, M. Geiser, Z. Kraus, D. Hauck, V. Kuban”; 1 male and 2 females, “Laos–NE: Houa Phan Province, Ban Saluei to Phou Pane Mt., 20.13N/ 104.00E, 1350–1500 m, 1–16. VI. 2009, M. Brancucci Leg.”, and “NHMB Basel, NMPC Prague Laos 2009 Expedition: M. Brancucci, M. Geiser, Z. Kraus, D. Hauck, V. Kuban”; 3 females, “Laos–NE: Xieng Khouang Province, 30 km NE Phonsaven: Ban Na Lam to Phou Sane Mt., 19.37–38N/ 103.20E, 1300–1500 m, 10.–30.V. 2009, M. Brancucci Leg.”, and “NHMB Basel, NMPC Prague Laos 2009 Expedition: M. Brancucci, M. Geiser, Z. Kraus, D. Hauck, V. Kuban”; 2 female, “Laos–NE: Houa Phan Province, Ban Saluei to Phou Pane Mt., 20.12–13N/ 103.595–104.01E, 1340–1870 m, 10. V.–16. VI. 2009, M. Brancucci and local coll. Leg.”, and “NHMB Basel, NMPC Prague Laos 2009 Expedition: M. Brancucci, M. Geiser, Z. Kraus, D. Hauck, V. Kuban”; 1 male 6 female, “Laos–NE: Xieng Khouang Province, Phonsaven (30 km NE), Phou Sane Mt., 19.37–38N/ 103.20E, 1400–1500 m, 10.–30.V. 2009, Z. Kraus Leg.”, and “NHMB Basel, NMPC Prague Laos 2009 Expedition: M. Brancucci, M. Geiser, Z. Kraus, D. Hauck, V. Kuban”; 1 females, “Laos–N: (Oudom Xai), 17 km, NEE of Oudom Xai, 20.45N/ 102.09E, 1–9. V. 2002, V. Kuban leg.”; 7 females, “Laos–NE: Xieng Khouang Province, Phonsaven (30 km NE), Phou Sane Mt., 19.37–38N/ 103.20–21E, 1400–1700 m, 10.–30.V. 2009, D. Hauck Leg.”, and “NHMB Basel, NMPC Prague Laos 2009 Expedition: M. Brancucci, M. Geiser, Z. Kraus, D. Hauck, V. Kuban”; 1 female, “Laos–NE: Houa Phan Province, Ban Saluei to Phou Pane Mt., 20.11–13N/ 103.59–104.01E, 1300–1900 m, 9–17.VI. 2009, Michael Geiser Leg.”, and “NHMB Basel, NMPC Prague Laos 2009 Expedition: M. Brancucci, M. Geiser, Z. Kraus, D. Hauck, V. Kuban”, all in NHMB except two males and ten females in MNHN and SCAU, respectively. 4 female, “Lao, Phongsaly Prov., 21°41–2'N /102°6–8'E, 28. v.–20. vi. 2003, Phongsaly env., 1500 m, Pacholátko leg.”, and “Collection Naturhistorisches Museum Basel”, all in NHMB except one in MNHN; 1 female, ibid, except “Pacholátko leg” replaced by “Brancucci leg.”, in NHMB.

#### Etymology.

Referring to the character of elytral carination.

#### Distribution.

Northern Laos (Fig. 1).

### Orthogonius macrophthalmus

Tian & Deuve sp. n.

http://zoobank.org/47B37F0F-48CD-4F1A-8E8A-398172CF0AA4

http://species-id.net/wiki/Orthogonius_macrophthalmus

Figs 1 , 5

#### Diagnosis.

Large sized, elytra broader and more ovate; head stout, as long as wide, covered with dense intricate wrinkles mixed with punctures; eyes very large and strongly prominent; labrum round at front; pronotum wide and distinctly transverse; whole lateral expanded margins distinctly reflexed; elytra carinate only on basal 1/3 of interval 7, intervals 4–6 normal; elytral apex distinctly denticulate.

Length: 16.0 mm; width: 6.5 mm. Habitus as in Fig. 5.

#### Description.

Head, pronotum and elytra black, antennomeres 1–4, clypeus, mandibles dark brown, palps, tarsi, and rest of antennae red brown.

Macrosculpture: Head densely wrinkled, punctures dense on frons and sparser on vertex and neck; pronotum glabrous and smooth, impunctate; elytra finely punctate on all intervals; underside smooth and glabrous.

Microsculpture: Engraved meshes densely isodiametric on elytra, somewhat transverse on head and pronotum.

Head stout, as long as wide,eyes very large, strongly prominent, frons and vertex convex, frontal impressions not well defined, wide, covered with punctures and wrinkles, not extending over level of middle eyes or anterior supraorbital pores; clypeus bisetose, base rugose; labrum sexsetose, front broadly convex; mandibles rather short, widened at base, median tooth distinct; ligula slightly dilated, bisetose at apex; maxillary palps rather stout, subcylindrical, maxillary palpomere 3 distinctly longer than 4, both glabrous, except for palpomere 3 with two setae at apex; labial palpomeres stout, palpomere 3 expanded, less twice as long as wide; palpomere 2 cylindrical, as long as palpomere 3, bisetose on inner margin, and with two or three additional setae at subapex and apex; palpiger asetose, mentum and submentum each with one pair of long setae; mentum without median tooth. Antennae long, beyond basal 1/3 of elytra; pubescent from basal 1/3 of antennomere 4, antennomere 3 as long as 4.

Pronotum strongly transverse, PW/PL=1.71, disc moderately convex; sides evenly rounded, widest at about middle, both basal and fore margins beaded, lateral expanded margins well defined and smooth, narrowest near fore angle, then gradually widened towards base, moderately reflexed throughout; fore and hind angles rounded; both fore and hind transversal impressions marked, basal foveae small; median line clear.

Elytra broadly ovate, EL/EW=1.50, moderately convex, basal border complete; sides slightly dilated at middle, hardly parallel-sided; striae deep, intervals distinctly convex, intervals subequal in width in middle; apex broadly and roundly truncate, outer angle rounded, inner angle nearly rectangular, denticulate; carinate on 2/3 of intervals 7 from base, other intervals normal; interval 3 with only the basal and middle setiferous pores, subapical pore absent.

Prosternal process faintly bordered at apex.

Legs slender; middle and hind coxae glabrous in median portion; fore tibia slightly dilated at apex, outer angle obtuse, apex slightly and obliquely truncate, outer margin not deeply sinuate, subserrate; middle and hind tibiae hardly dilated at apex; hind tibia with apical spurs long and sharp; hind femur slightly expanded medially, with two long setae posteriorly; hind tarsomeres 1 and 3 much longer than 2 and 4, respectively, length ratio of hind tarsomeres 1–4 as 4.3: 2.6: 1.6: 1.0; tarsomere 4 asymmetrically an deeply emarginate at apex, lobe about 1/3 as long as length; claws moderately pectinate.

**Male.** Unknown.

#### Remarks.

This new species is close to *Orthogonius carinatus* sp. n., but differs in having larger and strongly prominent eyes; wider and distinctly transverse pronotum (narrower and slightly transverse in *Orthogonius carinatus* sp. n.); short and broader elytra (slender in *Orthogonius carinatus* sp. n.), and only interval 7 in female carinate (bases of intervals 4–6 as well as interval 7 carinate in *Orthogonius carinatus* sp. n.).

#### Material examined.

Holotype: female, labeled: “I.R.Sc.N.B / Vietnam: Pia-Oac Mt., 22.36N/ 105.53E; pine forest (light trap), 03–VIII–2010; I.G.31.668, leg. J. Constant & P. Limbourg”, in IRSNB.

#### Etymology.

The name of the new species refers to its large and markedly prominent eyes.

#### Distribution.

Northern Vietnam (Fig. 1).

## Supplementary Material

XML Treatment for Orthogonius lancangjiang

XML Treatment for Orthogonius euthyphallus

XML Treatment for Orthogonius euthyphallus bolavenensis

XML Treatment for Orthogonius carinatus

XML Treatment for Orthogonius macrophthalmus
